# Clinical nursing pathway improves the nursing satisfaction in patients with acute cerebral hemorrhage: A randomized controlled trial protocol

**DOI:** 10.1097/MD.0000000000022989

**Published:** 2020-10-30

**Authors:** Su Fu, Hui Han, Chaofeng Fan, Yan Jiang

**Affiliations:** a Department of Neurological comprehensive ward; b Department of Nursing, West China Hospital of Sichuan University/West China Nursing College, Sichuan, China.

**Keywords:** Barthel index score, cerebral hemorrhage, clinical nursing pathway, function, protocol

## Abstract

**Background:**

Cerebral hemorrhage (CH) is a very common cerebrovascular disorder in clinical practice. More and more studies reported that proper nursing care could promote the rate of treatment, and improve the prognosis after treatment. Clinical nursing pathway (CNP) refers to original nursing mode with good quality, outstanding efficiency, and low treatment spending. Few articles have reported the effect of CNP in patients with acute CH. The program is in urgent need of convinced evidence to prove the reliability. Thus, we perform this randomized controlled trial protocol and hypothesize that CNP is associated with improved outcomes and nursing satisfaction, reduced adverse reactions in patients with acute CH.

**Method:**

It is a single-center randomized controlled study to be conducted from October 2020 to October 2021. It was admitted via the Ethics Committee of the West China Hospital of Sichuan University (0038842/121). Eighty patients meet diagnostic standards for CH are included. The study group receives the clinical nursing path model. In the control group, patients receive the routine care before and after taking to the hospital. The main outcome contains the Barthel index score, the patient's degree of satisfaction about care, the length of hospital stay, and the risk of complications such as infection, bedsores and gastrointestinal function between the 2 groups. Six months after admission, the functional independence measure and Fugl Meyer score are recorded. All data are analyzed by the IBM SPSS Statistics, version 20 (IBM Corp., Armonk, NY edition).

**Results:**

Table 1 shows the clinical outcomes between groups.

**Conclusion:**

CNP may improve the clinical outcomes for patients with acute CH and have a significant value in actual applications.

**Trial registration number:**

researchregistry6061

## 1 Introduction

Cerebral hemorrhage (CH) is a very common cerebrovascular disorder in clinical practice.^[1,2]^ Patients with this disease usually have trouble walking, speaking, and numbness on the face, arms or legs, which greatly affects their health-related quality.^[3,4]^ In addition, it results in the high morbidity and mortality in CH patients. It is estimated that the mortality rate of such patients can achieve 40% to 50%, and 75% of these patients have little chance living independently 1 year after the stroke.^[5]^ Due to the rise in the senior citizen population, the prevalence and occurring chance are likely to climb. Hematoma size and hematoma enlargement are still not negligible predictors of negative prognosis. Though early surgery can remove hematomas and refine secondary brain damage, aggressive drug treatment also stands a valid method to hold back the enlargement and rebreeding of intracranial hemorrhage.^[6,7]^

Besides the various treatments, more and more studies reported that proper nursing care can significantly improve the treatment rate and the prognosis.^[8,9]^ The role of nurses and the influence of nursing interventions on health consequences are rarely described and analyzed in recent guidelines. At present, with the rapid progress of medical technology, the nursing mode must be updated accordingly. Clinical nursing pathway (CNP), is an original nursing mode with good quality, outstanding efficiency and low treatment spending.^[10,11]^ Xu et al^[12]^ reported that for patients with malignant tumors, utilizing mental health CNP can reduce the likelihood of generating suicidal idea and positively affect patients’ life. Few articles have reported the effect of CNP in patients with acute CH. The program is in urgent need of convinced evidence to prove the reliability. Thus, we perform this randomized controlled trial protocol and hypothesize that CNP is associated with improved outcomes and nursing satisfaction, reduced adverse reactions in patients with acute CH.

## 2 Materials and methods

### 2.1 Study design

It is a single-center randomized controlled study to be conducted from October 2020 to October 2021. This study is performed according to the SPIRIT Checklist of randomized researches. It was admitted via the Ethics Committee of the West China Hospital of Sichuan University (0038842/121), and it has been registered in the research registry (researchregistry6061).

### 2.2 Subjects and randomization

There are 80 patients meeting the diagnostic standard for acute CH. Symptoms contain sudden clinical symptoms such as getting dizzy, bad physical condition and speech, getting sudden falls and coma, and the presence of fresh high-density shadows confirmed by emergency computed tomography (CT). In the random envelope, a random number is assigned to whole patients through the random-number table, and the distribution result is invisible. Patients are assigned randomly to study group (CNP, including forty samples) and control group (routine nursing mode, including 40 samples).

### 2.3 Intervention

The research team receives a clinical care pathway model. The model includes:

(1) Patients admitted from the green channel are stratified before admission. They receive rapid diagnosis, perform accurate examinations, apply vital signs support and emergency medicine, and quickly transferred to relevant experts;
(2) General professional courses of hospital care.

On the day of admission, the nursing team formulates the nursing path in combination with the patient's actual situation and domestic and foreign standards, and supplies and modifies the nursing content according to the actual operational problems encountered. Instruct patients to complete medication and care plan, monitor diet. The nursing team evaluates the patient's general condition and preventive measures for seizures and other adverse events are conducted using emergency equipment and proper medication. 2 to 7 days after admission, the nurse on duty assesses the patient's condition and limbs, and examines the skin and pupil. On the 8th and 14th day after admission, the nurse guides and assists patients with early rehabilitation education and training. After discharge, the nursing team evaluates the nursing path planning results and objectively records the deviations.

### 2.4 Control group

The control group receives routine nursing before and after admission. Patients are mainly admitted to the hospital through the emergency department, outpatient registration or emergency green channel. To determine the severity of the disease, emergency CT examination is performed on the patient upon admission. To prevent complications, indwelling of central vein, intranasal gastrointestinal nutritional support, catheterization, continuous monitoring of oxygen, vital signs, physical cooling, alcohol sponge bath, and antipyretic drug therapy may be considered. The nurse gives the patient medication and prepares for the operation according to the doctor's instructions. Explain the situation to the family and inform them that the situation may change.

### 2.5 Outcomes

The main outcome contains the Barthel index score,^[13]^ the patient's degree of satisfaction about care, the length of hospital stay, and the risk of complications such as infection, bedsores and gastrointestinal function between the 2 groups.

### 2.6 Statistical analysis

All data are analyzed by the IBM SPSS Statistics for Windows, version 20 (IBM Corp., Armonk, NY). Afterwards, all the data are described with appropriate characteristics such as mean, median, standard deviation as well as percentage. Continuous and categorical variables are analyzed using χ2-tests and independent *t* tests, respectively. Intention-to-treat analysis is used for the outcome assessments. When *P* value < .05, it is considered to be significant in statistics.

## 3 Results

Table 1 shows the clinical outcomes between groups.

**Table 1:**
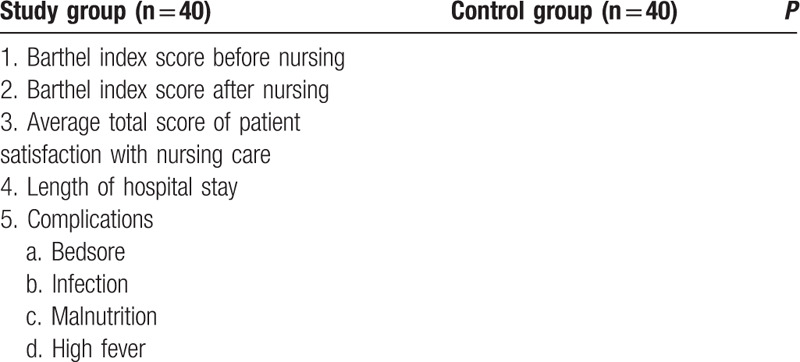
The clinical outcomes between groups.

## 4 Discussion

Acute CH, hypertension, atherosclerosis, coronary heart disease, diabetes, and other long-term diseases are closely related.^[14–16]^ Effective treatment with valid nursing care are the key to improve the prognosis and curative rate.^[17]^ The normal nursing model has some defects, because of the backward concept, 1-size-fits-all, cannot fully adapt to the development of the illness. Consequently, the normal nursing model is not ideal in meeting the most critical clinical needs of patients. CNP is an interdisciplinary, deeply integrated and progressive modern nursing model that emphasizes pre-admission hierarchy and comprehensive hospital nursing courses to improve nursing services for patients.^[18]^ This method is designed to greatly improve the quality of care, with patient-centered roles, while nursing and medical personnel focus on their best efforts to meet the needs of patients. The model can be presented as a table to help patients understand the content of self-care plan and the implication of active participation in disease rehabilitation.

## 5 Conclusion

CNP may improve the clinical outcomes for patients with acute CH and have a significant value in actual applications.

## Author contributions

Yan Jiang planned the study design. Chaofeng Fan and Hui Han reviewed the study protocol and collected data. Su Fu finished the manuscript. All of the authors approved the article and there is no conflict of interest.

**Formal analysis:** Hui Han, Chaofeng Fan.

**Funding acquisition:** Yan Jiang.

**Investigation:** Chaofeng Fan.

**Methodology:** Chaofeng Fan.

**Writing – original draft:** Su Fu.

**Writing – review & editing:** Hui Han, Chaofeng Fan.
